# *N*-(3-Methyl­phen­yl)pyrimidin-2-amine

**DOI:** 10.1107/S1600536810033301

**Published:** 2010-08-28

**Authors:** Edura Badaruddin, Zaharah Aiyub, Zanariah Abdullah, Seik Weng Ng, Edward R. T. Tiekink

**Affiliations:** a Department of Chemistry, University of Malaya, 50603 Kuala Lumpur, Malaysia

## Abstract

Two independent mol­ecules comprise the asymmetric unit in the title compound, C_11_H_11_N_3_. These differ in terms of the relative orientations of the aromatic rings: the first is somewhat twisted, while the second is approximately planar [dihedral angles between the pyrimidine and phenyl rings = 39.00 (8) and 4.59 (11)°]. The mol­ecules also form distinct patterns in their hydrogen bonding. The first independent mol­ecule forms centrosymmetric dimers featuring an eight-membered {HNCN}_2_ synthon. The second independent mol­ecule forms an N—H⋯N hydrogen bond with the other pyrimidine N atom of the first mol­ecule. Thereby, tetra­meric aggregates are formed. These associate *via* C—H⋯N and C—H⋯π inter­actions, consolidating the crystal packing.

## Related literature

For background to the fluorescence properties of compounds related to the title compound, see: Kawai *et al.* (2001 ▶); Abdullah (2005 ▶). For the structures of related pyrimidine amine derivatives, see: Badaruddin *et al.* (2009 ▶); Fairuz *et al.* (2010 ▶).
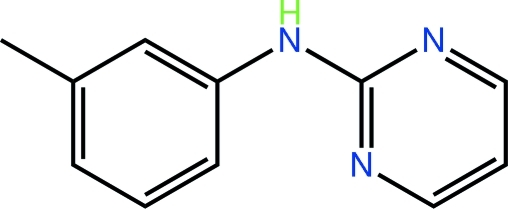


## Experimental

### 

#### Crystal data

C_11_H_11_N_3_
*M* *_r_* = 185.23
Triclinic, 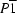

*a* = 9.4461 (10) Å
*b* = 10.0946 (11) Å
*c* = 11.6266 (13) Å
α = 80.401 (1)°
β = 82.745 (2)°
γ = 66.005 (1)°
*V* = 996.55 (19) Å^3^
*Z* = 4
Mo *K*α radiation
μ = 0.08 mm^−1^
*T* = 293 K
0.20 × 0.20 × 0.10 mm

#### Data collection

Bruker SMART APEX diffractometer
9569 measured reflections
4539 independent reflections
2881 reflections with *I* > 2σ(*I*)
*R*_int_ = 0.035

#### Refinement

*R*[*F* ^2^ > 2σ(*F* ^2^)] = 0.051
*wR*(*F* ^2^) = 0.167
*S* = 1.02
4539 reflections
264 parameters
2 restraints
H atoms treated by a mixture of independent and constrained refinement
Δρ_max_ = 0.24 e Å^−3^
Δρ_min_ = −0.22 e Å^−3^

### 

Data collection: *APEX2* (Bruker, 2009 ▶); cell refinement: *SAINT* (Bruker, 2009 ▶); data reduction: *SAINT*; program(s) used to solve structure: *SHELXS97* (Sheldrick, 2008 ▶); program(s) used to refine structure: *SHELXL97* (Sheldrick, 2008 ▶); molecular graphics: *ORTEP-3* (Farrugia, 1997 ▶), *DIAMOND* (Brandenburg, 2006 ▶) and *Qmol* (Gans & Shalloway, 2001 ▶); software used to prepare material for publication: *publCIF* (Westrip, 2010 ▶).

## Supplementary Material

Crystal structure: contains datablocks global, I. DOI: 10.1107/S1600536810033301/bt5328sup1.cif

Structure factors: contains datablocks I. DOI: 10.1107/S1600536810033301/bt5328Isup2.hkl

Additional supplementary materials:  crystallographic information; 3D view; checkCIF report

## Figures and Tables

**Table 1:** Hydrogen-bond geometry (Å, °) *Cg*1 and *Cg*2 are the centroids of the N4,N5,C12–C15 and C5–C10 rings, respectively.

*D*—H⋯*A*	*D*—H	H⋯*A*	*D*⋯*A*	*D*—H⋯*A*
N3—H3⋯N2^i^	0.86 (1)	2.19 (1)	3.0377 (19)	170 (2)
N6—H6⋯N1	0.87 (1)	2.45 (1)	3.2391 (19)	151 (2)
C6—H6a⋯N1	0.93	2.55	2.961 (2)	107
C17—H17⋯N4	0.93	2.28	2.886 (3)	123
C11—H11a⋯*Cg*1^ii^	0.96	2.96	3.766 (2)	143
C15—H15⋯*Cg*2^iii^	0.93	2.82	3.620 (2)	144

Symmetry codes: (i) 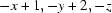; (ii) 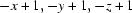; (iii) 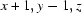.

**Fig. 1.**
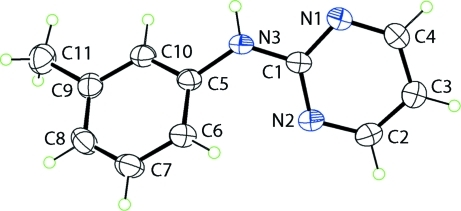
The molecular structure of the first independent molecule in (I) showing the atom-labelling scheme and displacement ellipsoids at the 50% probability level.

**Fig. 2.**
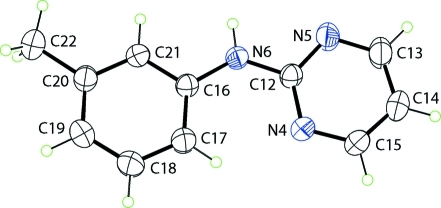
The molecular structure of the second independent molecule in (I) showing the atom-labelling scheme and displacement ellipsoids at the 50% probability level.

**Fig. 3.**
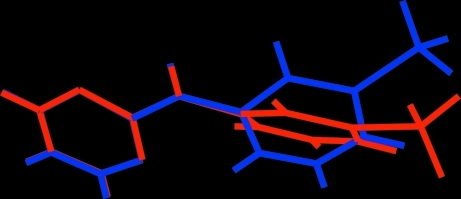
Overlay diagram of the first independent molecule (shown in red) and the second independent molecule (shown in blue).

**Fig. 4.**
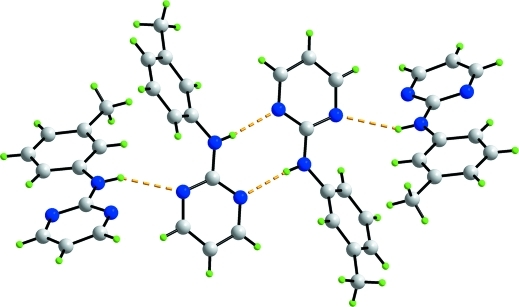
Supramolecular tetrameric aggregate in (I) mediated by N–H···N hydrogen bonding, shown as orange dashed lines.

**Fig. 5.**
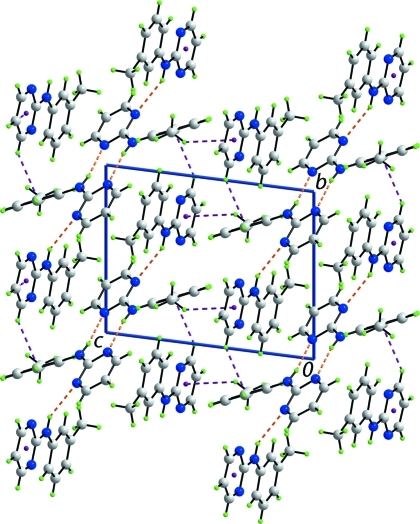
Unit-cell contents for (I) shown in projection down the a axis. The N–H···N hydrogen bonding and C–H···π contacts are shown as orange and purple dashed lines, respectively.

## supplementary crystallographic information

### Comment

The title compound, (I), was investigated as a continuation of structural studies of pyrimidine derivatives related to the title compound (Badaruddin *et al.*, 2009; Fairuz *et al.*, 2010). Interest in these compounds relates to the fluorescence properties of related compounds (Kawai *et al.* 2001; Abdullah, 2005).

Two independent molecules of comprise the asymmetric unit of (I), Figs 1 and 2. Whereas the pyrimidine and amine residues are super-imposable in the two molecules, there is a large difference in the relative orientations of the tolyl groups, Fig. 3. This is seen in the marked difference in the dihedral angles formed between the pyrimidine and benzene rings of 39.00 (8) ° for the first independent molecule and 4.59 (11) ° for the second. Hence, while the first molecule is significantly twisted about the N3–C5 bond [the C1–N3–C5–C6 torsion angle = 37.2 (3) °], the second molecule is essentially planar [r.m.s. deviation of the 14 non-hydrogen atoms = 0.046 Å; the equivalent C12–N6–C16–C17 torsion angle is 1.1 (3) °]. It is noted that each of the N2 and N4 atoms forms a significant intramolecular C–H···N contact and that the contact formed in the second independent molecule is significantly shorter, Table 1.

Over and above the conformational differences between the independent molecules, they form quite distinct patterns in their intermolecular contacts. Thus, centrosymmetrically related molecules of the first independent species associate *via* an eight-membered {···HNCN}_2_ synthon, Table 1. The pyrimdine atom not participating in this synthon accepts an N–H hydrogen bond from the second independent molecule. In this way, tetrameric supramolecular aggregates are formed, Fig. 4. Therefore, while each of the nitrogen atoms of the first independent molecule participates in intermolecular interactions, only the amine-nitrogen of the second molecule forms a significant intermolecular interaction. The tetrameric aggregates are consolidated into the three-dimensional structure by C–H···π interactions, Fig. 5 and Table 1.

### Experimental

2-Chloropyrimidine (3.1701 g, 30 mmol) was mixed with 3-methylaniline (3 ml, 30 mmol) along with several drops of ethanol. The mixture was heated at 423–433 K for 5 h. The organic phase was dried over sodium sulfate; the evaporation of the solvent gave the colourless crystals.

### Refinement

Carbon-bound H-atoms were placed in calculated positions (C—H 0.93 to 0.96 Å) and were included in the refinement in the riding model approximation, with *U*_iso_(H) set to 1.2 to 1.5*U*_equiv_(C). The N-bound H-atoms were located in a difference Fourier map and were were refined with a distance restraint of N–H = 0.86±0.01 Å, and with *U*_iso_(H) = 1.2*U*_eq_(N).

### Crystal data

**Table:**

C_11_H_11_N_3_	*Z* = 4
*M**_r_* = 185.23	*F*(000) = 392
Triclinic, *P*1	*D*_x_ = 1.235 Mg m^−^^3^
Hall symbol: -P 1	Mo *K*α radiation, λ = 0.71073 Å
*a* = 9.4461 (10) Å	Cell parameters from 2493 reflections
*b* = 10.0946 (11) Å	θ = 4.4–24.7°
*c* = 11.6266 (13) Å	µ = 0.08 mm^−^^1^
α = 80.401 (1)°	*T* = 293 K
β = 82.745 (2)°	Block, colourless
γ = 66.005 (1)°	0.20 × 0.20 × 0.10 mm
*V* = 996.55 (19) Å^3^	

### Data collection

**Table:**

Bruker SMART APEX diffractometer	2881 reflections with *I* > 2σ(*I*)
Radiation source: fine-focus sealed tube	*R*_int_ = 0.035
graphite	θ_max_ = 27.5°, θ_min_ = 1.8°
ω scans	*h* = −11→12
9569 measured reflections	*k* = −12→13
4539 independent reflections	*l* = −15→15

### Refinement

**Table:**

Refinement on *F*^2^	Secondary atom site location: difference Fourier map
Least-squares matrix: full	Hydrogen site location: inferred from neighbouring sites
*R*[*F*^2^ > 2σ(*F*^2^)] = 0.051	H atoms treated by a mixture of independent and constrained refinement
*wR*(*F*^2^) = 0.167	*w* = 1/[σ^2^(*F*_o_^2^) + (0.0942*P*)^2^ + 0.0196*P*] where *P* = (*F*_o_^2^ + 2*F*_c_^2^)/3
*S* = 1.02	(Δ/σ)_max_ = 0.001
4539 reflections	Δρ_max_ = 0.24 e Å^−^^3^
264 parameters	Δρ_min_ = −0.22 e Å^−^^3^
2 restraints	Extinction correction: *SHELXL97* (Sheldrick, 2008), Fc^*^=kFc[1+0.001xFc^2^λ^3^/sin(2θ)]^-1/4^
Primary atom site location: structure-invariant direct methods	Extinction coefficient: 0.016 (4)

### Special details

**Table:**

Geometry. All e.s.d.'s (except the e.s.d. in the dihedral angle between two l.s. planes) are estimated using the full covariance matrix. The cell e.s.d.'s are taken into account individually in the estimation of e.s.d.'s in distances, angles and torsion angles; correlations between e.s.d.'s in cell parameters are only used when they are defined by crystal symmetry. An approximate (isotropic) treatment of cell e.s.d.'s is used for estimating e.s.d.'s involving l.s. planes.
Refinement. Refinement of *F*^2^ against ALL reflections. The weighted *R*-factor *wR* and goodness of fit *S* are based on *F*^2^, conventional *R*-factors *R* are based on *F*, with *F* set to zero for negative *F*^2^. The threshold expression of *F*^2^ > σ(*F*^2^) is used only for calculating *R*-factors(gt) *etc*. and is not relevant to the choice of reflections for refinement. *R*-factors based on *F*^2^ are statistically about twice as large as those based on *F*, and *R*- factors based on ALL data will be even larger.

### Fractional atomic coordinates and isotropic or equivalent isotropic displacement parameters (Å^2^)

**Table:**

	*x*	*y*	*z*	*U*_iso_*/*U*_eq_	
N1	0.78783 (16)	0.67552 (15)	0.14005 (12)	0.0480 (4)	
N2	0.71434 (16)	0.88285 (15)	−0.00792 (12)	0.0511 (4)	
N3	0.53298 (16)	0.84550 (16)	0.12674 (12)	0.0499 (4)	
H3	0.4675 (17)	0.9178 (15)	0.0846 (14)	0.055 (5)*	
N4	1.05919 (19)	0.15235 (17)	0.35892 (15)	0.0635 (4)	
N5	1.0388 (2)	0.39503 (18)	0.35802 (17)	0.0721 (5)	
N6	0.84789 (17)	0.35680 (16)	0.28605 (12)	0.0482 (4)	
H6	0.810 (2)	0.4518 (10)	0.2708 (16)	0.060 (6)*	
C1	0.68337 (19)	0.79917 (17)	0.08648 (13)	0.0425 (4)	
C2	0.9333 (2)	0.6381 (2)	0.09545 (16)	0.0556 (5)	
H2	1.0093	0.5542	0.1308	0.067*	
C3	0.9782 (2)	0.7156 (2)	0.00069 (17)	0.0624 (5)	
H3A	1.0811	0.6871	−0.0285	0.075*	
C4	0.8615 (2)	0.8385 (2)	−0.04876 (16)	0.0574 (5)	
H4	0.8872	0.8931	−0.1142	0.069*	
C5	0.46706 (18)	0.78759 (16)	0.22835 (14)	0.0425 (4)	
C6	0.5414 (2)	0.73374 (18)	0.33221 (14)	0.0485 (4)	
H6A	0.6400	0.7313	0.3370	0.058*	
C7	0.4663 (2)	0.6841 (2)	0.42779 (15)	0.0574 (5)	
H7	0.5158	0.6464	0.4973	0.069*	
C8	0.3197 (2)	0.6892 (2)	0.42267 (16)	0.0580 (5)	
H8	0.2718	0.6544	0.4885	0.070*	
C9	0.2421 (2)	0.74531 (17)	0.32074 (15)	0.0494 (4)	
C10	0.31905 (19)	0.79320 (17)	0.22345 (15)	0.0458 (4)	
H10	0.2700	0.8297	0.1537	0.055*	
C11	0.0801 (2)	0.7550 (2)	0.3143 (2)	0.0685 (6)	
H11A	0.0143	0.8042	0.3771	0.103*	
H11B	0.0818	0.6583	0.3207	0.103*	
H11C	0.0408	0.8086	0.2409	0.103*	
C12	0.9877 (2)	0.29674 (18)	0.33629 (14)	0.0464 (4)	
C13	1.1733 (3)	0.3396 (3)	0.4079 (2)	0.0846 (7)	
H13	1.2123	0.4041	0.4260	0.102*	
C14	1.2576 (2)	0.1932 (3)	0.4342 (2)	0.0733 (6)	
H14	1.3522	0.1572	0.4682	0.088*	
C15	1.1951 (2)	0.1040 (2)	0.4078 (2)	0.0715 (6)	
H15	1.2494	0.0036	0.4245	0.086*	
C16	0.75431 (19)	0.29091 (18)	0.25653 (14)	0.0444 (4)	
C17	0.7885 (3)	0.1426 (2)	0.2762 (2)	0.0712 (6)	
H17	0.8790	0.0783	0.3110	0.085*	
C18	0.6869 (3)	0.0911 (2)	0.2434 (3)	0.0877 (8)	
H18	0.7101	−0.0087	0.2572	0.105*	
C19	0.5535 (3)	0.1820 (2)	0.1916 (2)	0.0752 (6)	
H19	0.4879	0.1438	0.1696	0.090*	
C20	0.5161 (2)	0.3304 (2)	0.17184 (17)	0.0576 (5)	
C21	0.6174 (2)	0.38285 (19)	0.20499 (15)	0.0511 (4)	
H21	0.5930	0.4829	0.1923	0.061*	
C22	0.3675 (3)	0.4331 (3)	0.1169 (2)	0.0877 (8)	
H22A	0.2841	0.4052	0.1508	0.132*	
H22B	0.3793	0.4284	0.0343	0.132*	
H22C	0.3446	0.5312	0.1307	0.132*	

### Atomic displacement parameters (Å^2^)

**Table:**

	*U*^11^	*U*^22^	*U*^33^	*U*^12^	*U*^13^	*U*^23^
N1	0.0448 (8)	0.0418 (8)	0.0489 (8)	−0.0109 (6)	−0.0080 (6)	0.0043 (6)
N2	0.0493 (9)	0.0483 (8)	0.0462 (8)	−0.0143 (7)	−0.0032 (6)	0.0064 (6)
N3	0.0418 (8)	0.0474 (8)	0.0475 (8)	−0.0107 (7)	−0.0077 (6)	0.0143 (6)
N4	0.0607 (10)	0.0478 (9)	0.0775 (11)	−0.0105 (8)	−0.0246 (8)	−0.0092 (8)
N5	0.0761 (12)	0.0559 (10)	0.0930 (13)	−0.0330 (9)	−0.0399 (10)	0.0119 (9)
N6	0.0512 (8)	0.0402 (8)	0.0525 (8)	−0.0177 (7)	−0.0127 (7)	0.0020 (6)
C1	0.0456 (9)	0.0385 (8)	0.0404 (8)	−0.0144 (7)	−0.0086 (7)	0.0015 (6)
C2	0.0466 (10)	0.0502 (10)	0.0570 (11)	−0.0077 (8)	−0.0090 (8)	0.0016 (8)
C3	0.0473 (10)	0.0673 (12)	0.0577 (11)	−0.0125 (9)	0.0037 (8)	−0.0002 (9)
C4	0.0563 (11)	0.0601 (11)	0.0470 (10)	−0.0200 (9)	0.0022 (8)	0.0042 (8)
C5	0.0454 (9)	0.0329 (8)	0.0435 (8)	−0.0112 (7)	−0.0037 (7)	0.0006 (6)
C6	0.0511 (10)	0.0465 (9)	0.0459 (9)	−0.0178 (8)	−0.0091 (7)	0.0002 (7)
C7	0.0656 (12)	0.0569 (11)	0.0439 (9)	−0.0205 (9)	−0.0103 (8)	0.0045 (8)
C8	0.0694 (13)	0.0531 (11)	0.0475 (10)	−0.0263 (10)	0.0060 (9)	0.0025 (8)
C9	0.0498 (10)	0.0371 (9)	0.0579 (10)	−0.0154 (8)	0.0013 (8)	−0.0055 (7)
C10	0.0477 (10)	0.0379 (9)	0.0472 (9)	−0.0132 (7)	−0.0069 (7)	−0.0002 (7)
C11	0.0579 (12)	0.0648 (13)	0.0832 (14)	−0.0285 (10)	0.0022 (10)	−0.0046 (11)
C12	0.0487 (10)	0.0473 (10)	0.0418 (8)	−0.0184 (8)	−0.0072 (7)	0.0003 (7)
C13	0.0835 (16)	0.0721 (15)	0.1102 (19)	−0.0391 (13)	−0.0499 (14)	0.0114 (13)
C14	0.0601 (13)	0.0768 (15)	0.0808 (14)	−0.0206 (11)	−0.0286 (11)	−0.0028 (11)
C15	0.0639 (13)	0.0549 (12)	0.0852 (15)	−0.0046 (10)	−0.0276 (11)	−0.0132 (10)
C16	0.0460 (9)	0.0450 (9)	0.0418 (8)	−0.0181 (8)	−0.0038 (7)	−0.0031 (7)
C17	0.0688 (13)	0.0477 (11)	0.1000 (16)	−0.0234 (10)	−0.0330 (12)	0.0043 (11)
C18	0.0877 (16)	0.0487 (12)	0.137 (2)	−0.0316 (12)	−0.0446 (15)	0.0017 (13)
C19	0.0666 (13)	0.0669 (14)	0.1044 (18)	−0.0318 (11)	−0.0199 (12)	−0.0187 (12)
C20	0.0494 (10)	0.0616 (12)	0.0615 (11)	−0.0174 (9)	−0.0082 (8)	−0.0150 (9)
C21	0.0509 (10)	0.0442 (9)	0.0555 (10)	−0.0140 (8)	−0.0091 (8)	−0.0072 (8)
C22	0.0640 (14)	0.0809 (16)	0.117 (2)	−0.0156 (12)	−0.0360 (13)	−0.0204 (14)

### Geometric parameters (Å, °)

**Table:**

N1—C2	1.328 (2)	C8—H8	0.9300
N1—C1	1.347 (2)	C9—C10	1.392 (2)
N2—C4	1.325 (2)	C9—C11	1.504 (3)
N2—C1	1.3470 (19)	C10—H10	0.9300
N3—C1	1.349 (2)	C11—H11A	0.9600
N3—C5	1.416 (2)	C11—H11B	0.9600
N3—H3	0.863 (9)	C11—H11C	0.9600
N4—C12	1.329 (2)	C13—C14	1.365 (3)
N4—C15	1.339 (2)	C13—H13	0.9300
N5—C13	1.328 (3)	C14—C15	1.351 (3)
N5—C12	1.336 (2)	C14—H14	0.9300
N6—C12	1.371 (2)	C15—H15	0.9300
N6—C16	1.404 (2)	C16—C17	1.381 (2)
N6—H6	0.872 (9)	C16—C21	1.388 (2)
C2—C3	1.367 (2)	C17—C18	1.378 (3)
C2—H2	0.9300	C17—H17	0.9300
C3—C4	1.376 (3)	C18—C19	1.364 (3)
C3—H3A	0.9300	C18—H18	0.9300
C4—H4	0.9300	C19—C20	1.378 (3)
C5—C10	1.384 (2)	C19—H19	0.9300
C5—C6	1.390 (2)	C20—C21	1.385 (2)
C6—C7	1.376 (2)	C20—C22	1.508 (3)
C6—H6A	0.9300	C21—H21	0.9300
C7—C8	1.374 (3)	C22—H22A	0.9600
C7—H7	0.9300	C22—H22B	0.9600
C8—C9	1.386 (3)	C22—H22C	0.9600
			
C2—N1—C1	115.41 (14)	C9—C11—H11B	109.5
C4—N2—C1	115.85 (14)	H11A—C11—H11B	109.5
C1—N3—C5	128.18 (13)	C9—C11—H11C	109.5
C1—N3—H3	116.6 (12)	H11A—C11—H11C	109.5
C5—N3—H3	115.1 (12)	H11B—C11—H11C	109.5
C12—N4—C15	115.44 (18)	N4—C12—N5	126.19 (16)
C13—N5—C12	115.19 (17)	N4—C12—N6	119.78 (16)
C12—N6—C16	130.75 (14)	N5—C12—N6	114.03 (15)
C12—N6—H6	114.5 (13)	N5—C13—C14	123.7 (2)
C16—N6—H6	114.7 (13)	N5—C13—H13	118.1
N1—C1—N2	125.62 (15)	C14—C13—H13	118.1
N1—C1—N3	119.24 (14)	C15—C14—C13	115.9 (2)
N2—C1—N3	115.13 (14)	C15—C14—H14	122.0
N1—C2—C3	123.91 (16)	C13—C14—H14	122.0
N1—C2—H2	118.0	N4—C15—C14	123.5 (2)
C3—C2—H2	118.0	N4—C15—H15	118.3
C2—C3—C4	115.81 (17)	C14—C15—H15	118.3
C2—C3—H3A	122.1	C17—C16—C21	118.40 (16)
C4—C3—H3A	122.1	C17—C16—N6	124.64 (16)
N2—C4—C3	123.38 (16)	C21—C16—N6	116.96 (15)
N2—C4—H4	118.3	C18—C17—C16	119.19 (19)
C3—C4—H4	118.3	C18—C17—H17	120.4
C10—C5—C6	119.87 (15)	C16—C17—H17	120.4
C10—C5—N3	117.36 (14)	C19—C18—C17	122.1 (2)
C6—C5—N3	122.68 (15)	C19—C18—H18	119.0
C7—C6—C5	118.69 (16)	C17—C18—H18	119.0
C7—C6—H6A	120.7	C18—C19—C20	119.86 (19)
C5—C6—H6A	120.7	C18—C19—H19	120.1
C8—C7—C6	121.37 (17)	C20—C19—H19	120.1
C8—C7—H7	119.3	C19—C20—C21	118.31 (18)
C6—C7—H7	119.3	C19—C20—C22	120.71 (18)
C7—C8—C9	120.87 (16)	C21—C20—C22	120.98 (18)
C7—C8—H8	119.6	C20—C21—C16	122.16 (17)
C9—C8—H8	119.6	C20—C21—H21	118.9
C8—C9—C10	117.80 (16)	C16—C21—H21	118.9
C8—C9—C11	121.64 (16)	C20—C22—H22A	109.5
C10—C9—C11	120.56 (17)	C20—C22—H22B	109.5
C5—C10—C9	121.37 (16)	H22A—C22—H22B	109.5
C5—C10—H10	119.3	C20—C22—H22C	109.5
C9—C10—H10	119.3	H22A—C22—H22C	109.5
C9—C11—H11A	109.5	H22B—C22—H22C	109.5
			
C2—N1—C1—N2	1.8 (3)	C15—N4—C12—N5	−0.2 (3)
C2—N1—C1—N3	−179.59 (15)	C15—N4—C12—N6	179.91 (17)
C4—N2—C1—N1	−1.1 (3)	C13—N5—C12—N4	−0.6 (3)
C4—N2—C1—N3	−179.76 (15)	C13—N5—C12—N6	179.29 (19)
C5—N3—C1—N1	6.3 (3)	C16—N6—C12—N4	3.7 (3)
C5—N3—C1—N2	−174.88 (16)	C16—N6—C12—N5	−176.17 (17)
C1—N1—C2—C3	−1.0 (3)	C12—N5—C13—C14	1.2 (4)
N1—C2—C3—C4	−0.3 (3)	N5—C13—C14—C15	−0.9 (4)
C1—N2—C4—C3	−0.4 (3)	C12—N4—C15—C14	0.5 (3)
C2—C3—C4—N2	1.1 (3)	C13—C14—C15—N4	0.0 (4)
C1—N3—C5—C10	−146.27 (17)	C12—N6—C16—C17	1.1 (3)
C1—N3—C5—C6	37.2 (3)	C12—N6—C16—C21	−179.49 (16)
C10—C5—C6—C7	1.3 (2)	C21—C16—C17—C18	0.4 (3)
N3—C5—C6—C7	177.79 (16)	N6—C16—C17—C18	179.8 (2)
C5—C6—C7—C8	−1.0 (3)	C16—C17—C18—C19	0.3 (4)
C6—C7—C8—C9	−0.3 (3)	C17—C18—C19—C20	−0.8 (4)
C7—C8—C9—C10	1.4 (3)	C18—C19—C20—C21	0.5 (3)
C7—C8—C9—C11	−178.53 (18)	C18—C19—C20—C22	−178.7 (2)
C6—C5—C10—C9	−0.2 (2)	C19—C20—C21—C16	0.3 (3)
N3—C5—C10—C9	−176.90 (15)	C22—C20—C21—C16	179.48 (19)
C8—C9—C10—C5	−1.1 (2)	C17—C16—C21—C20	−0.7 (3)
C11—C9—C10—C5	178.82 (16)	N6—C16—C21—C20	179.82 (16)

### Hydrogen-bond geometry (Å, °)

**Table:**

Cg1 and Cg2 are the centroids of the N4,N5,C12–C15 and C5–C10 rings, respectively.

**Table:**

*D*—H···*A*	*D*—H	H···*A*	*D*···*A*	*D*—H···*A*
N3—H3···N2^i^	0.86 (1)	2.19 (1)	3.0377 (19)	170.(2)
N6—H6···N1	0.87 (1)	2.45 (1)	3.2391 (19)	151.(2)
C6—H6a···N1	0.93	2.55	2.961 (2)	107
C17—H17···N4	0.93	2.28	2.886 (3)	123
C11—H11a···Cg1^ii^	0.96	2.96	3.766 (2)	143
C15—H15···Cg2^iii^	0.93	2.82	3.620 (2)	144

Symmetry codes: (i) −*x*+1, −*y*+2, −*z*;  (ii) −*x*+1, −*y*+1, −*z*+1; (iii) *x*+1, *y*−1, *z*.
